# Children of Single Fathers Created by Surrogacy: Psychosocial Adjustment Considerations and Implications for Research and Practice

**DOI:** 10.3390/children9111644

**Published:** 2022-10-28

**Authors:** Henrique Pereira

**Affiliations:** 1 Department of Psychology and Education, Faculty of Social and Human Sciences, University of Beira Interior, Pόlo IV, 6200-209 Covilhã, Portugal; hpereira@ubi.pt; 2 Research Centre in Sports Sciences, Health Sciences and Human Development (CIDESD), 5000-801 Vila Real, Portugal

**Keywords:** children, single fathers, surrogacy, psychosocial adjustment, mini review

## Abstract

The existence of single-father families formed by surrogacy is becoming a more visible reality, even though this type of family organization is still perceived with stigma and negative attitudes by more traditional sectors of society, because it raises some concerns regarding the psychosocial well-being of children who are born into single-fathers’ families via surrogacy, and in many cases, to gay single men who wish to become fathers. On the other hand, available research on the psychosocial well-being of these children is still very scarce and limited to a handful of Western countries. Hence, it is of utmost importance to examine studies that explore the psychosocial adjustment of these children. In this mini review, I show that all the studies revised demonstrate the good psychosocial adjustment of these children, and that they are as likely to flourish as children born into traditional families, even if they may find themselves exposed to prejudice and stigma. In conclusion, single fatherhood and surrogacy do not contribute to any adverse consequences to the children’s psychosocial development and adjustment, and there is no observed evidence to why single men, irrespective of their sexual orientation, should not be fathers via surrogacy. Finally, implications for future research and interventions are also discussed.

## 1. Introduction

As the occurrence of single-father families (defined as a group of one male parent and their children living together as a unit [1]) formed by surrogacy becomes more visible, and therefore, seen as a way of social modification that facilitates the debunking of more traditional understandings of the concept of family [2], more becomes known about those men who wish to be or who already are single fathers using surrogacy. Not only do they contest the current artificial reproductive technologies (ARTs) legal agenda, in which the legal body is primarily female, but they also must defy the challenges linked to the circumstance that their fatherhood will be illegal in most countries around the world [3]. Hence, single men seeking surrogacy, i.e., single men who are seeking to establish a contract in which a woman approves the gestation of a baby for them after ARTs, may have to deal with significant sociopolitical limitations, since even though there are some thirty-six countries worldwide with surrogacy regulations, the vast majority of them prohibits access to intended single fathers [4]. Nations that permit, or do not reject, single men from having access to ARTs via surrogacy are rarer and comprise Mexico, Georgia, Colombia, and the United States of America.

How many single-father families generated through surrogacy are there, is unknown and hard to determine. In the USA, for example, it is probable that over two-million men are single fathers, making up around seventeen percent of the single-parent population [5]. This also happens in the European Union, where around three percent of families comprise single-father families, although only a residual number will probably be attributable to surrogacy [6]. Notwithstanding, these men are usually encouraged by the aspiration to become biological fathers and to foster strong paternal relations, as well as by negative beliefs concerning the adoption structure in their home countries, and by positive examples of other single fathers who experienced surrogacy before them [7]. Regardless of sexual orientation, the majority of these men will express their wish to become single fathers since they value a genetic connection to their child and feel that surrogacy is securer than adoption because of the legal constrictions linked to the latter [8].

Under these circumstances, the impacts of parenthood plans, ARTs, stress during gestation and childbirth, and the transition to parenthood may all be complex and pose difficulties hard to manage, due to the lack of visibility and social support [9,10]. Additionally, surrogacy itself may pose specific challenges, because a single’s man choice of becoming a father through surrogacy (whether altruistic or commercial, traditional, or gestational) is often seen as an eccentricity and an exploration of the female body [11]. However, to break this level of complexities and moral judgement, many men choose to pursue gestational surrogacy, because the embryo is produced via the in vitro fertilization of gametes from an egg donor and the father, then being shifted to the surrogate’s uterus, creating no genetic relation between the baby and the surrogate [12].

The social narratives associated with how single fathers by surrogacy perceive themselves and are perceived by others, compels us to a new understanding and conceptualization of what it means to be a family, forming wider and more open views compared with more traditional family beliefs, making single fatherhood a multidimensional experience [13]. In this sense, ARTs cannot be seen as strict medical procedures, and surrogacy cannot be seen as a strict commercial transaction. Rather, they embody human existence and lead us to legitimate these men’s decisions, as well as their future relationships with their children, many times seen as inherently deficient due to the contingency of couplehood and parenthood [14].

Hence, examining the psychosocial health of children of single fathers created by surrogacy is of utmost importance. In fact, even if our understanding and validation of single-father families is linked with positive social attitudes, what is the impact on their children? Therefore, the purpose of this mini review is to present a summary of the investigation on single-father families formed by surrogacy, focusing on their psychosocial adjustment, and to explore some repercussions for research and practice.

## 2. Materials and Methods

### 2.1. Search Strategies

I conducted a literature search using APA PsycInfo, APA PsycArticles, APA PsycBooks, Psychology and Behavioral Sciences Collection, Web of Science, Medline, Academic Search Complete, EBSCOhost, and ERIC (from January 2017 to September 2022), according to the PRISMA report [15]. As search criteria, I used the following: single-father families, surrogacy, children, mental health, and psychosocial adjustment. The inclusion criteria were original articles including single-father families created by surrogacy containing children in their samples. No language restriction was applied. To obtain greater objectivity and validity in this mini review process, a protocol was developed, to define the objectives, research questions, eligibility criteria, research strategy and analysis of the quality of the included articles in the review itself.

### 2.2. Selection Criteria

After eliminating duplicates, studies were assessed for inclusion by title and abstract. Data were extracted using the following information: authorship, country, number and age of children participants, short description of methods, and main results. I limited the search to full text, published articles and human studies from the past five years. All studies that investigated the psychosocial adjustment of children of single-father families were considered.

## 3. Results

A total of 26 articles were organized from the article search. Following eliminating the duplicates, 14 articles were selected for inclusion, based on title and abstract review. After full-text study, only 8 articles met the inclusion criteria and were added to this mini review.

Table 1 shows the results of the mini review process of identifying and describing eight studies which demonstrate important contributions to the understanding of the psychosocial adjustment of children of single-fathers created by surrogacy, published from 2019 to 2022. Psychosocial adjustment indicators can be seen in the main results column and involve fewer behavioral problems, greater social support, lower anxiety and depression symptoms, higher attachment security, self-worth, and less conflict. Overall, the results show that children of single fathers created by surrogacy present well-adjusted psychosocial indicators, and that it is more a function of family processes than of family structure.

## 4. Discussion

### 4.1. Children of Single Fathers Created by Surrogacy: Psychosocial Adjustment Considerations

Most of the research conducted with children raised by single parents has focused on single mothers, leading to invisibilities that explain how single fathers, who are not legally protected to the degree that single mothers are in most countries, are kept out of the common field of vision and more exposed to stigmatization [3]. On the other hand, little research has been conducted with children born through surrogacy raised by single fathers, and most of the research tends to focus on the father’s experiences and not the child’s adjustment and psychosocial functioning, defined as the child’s capacity to adapt to the environment, assuming that he/she has enough psychological and social mechanisms to feel adapted, integrated, respond adequately to environmental demands, and achieve their personal goals with subjective well-being [24]. This has to do with several factors, including the lack of visibility of these families and the need for them to protect the children from the public eye due to the anticipation of stigma and discriminatory actions. However, some research is available regarding the child’s well-being, and I will summarize the main finding in this mini review.

Children are capable of developing quality attachments with any adult that interacts with them frequently [25], which means that single men are also able to form secure attachments with their surrogate-born children, even showing higher levels of motivation to do so [26]. In a study carried out with thirty-three surrogacy children with gay fathers, it was found that they demonstrated high levels of secure attachment to their fathers, not differing from the scores achieved by children raised in normative families, since these fathers were able to demonstrate responsiveness, parental warmth, and willingness to function as an attachment figure, hence being perceived as developmental safe havens [27]. In fact, some studies demonstrate that compared with natural conception parents, parents via surrogacy present more positive relational outcomes with their children, and their children are performing well in all areas of functionality [27], especially where spaces for conversation are created with children to explore their origins and, thereby promoting a better identity adjustment, especially during adolescence [19].

Causes related to behavioral and developmental adjustment amongst school aged children of heterosexual and gay single fathers via surrogacy have also been studied. The results indicate that irrespective of the father’s sexual orientation, children with a greater accepting and understanding of their surrogacy origin presented higher levels of self-worth, whereas those with lower comprehension of the surrogacy process, and lower comfort with their family constellation, externalized more behavioral problems [19]. Taken altogether, these results do not support the frequently thought notion that the mixture of surrogacy single fatherhood and conception is harmful to the child’s psychosocial adjustment. This is reinforced with similar findings conducted with single women who used surrogacy [28].

Another study revealed that the psychosocial adjustment of their children was more the result of family processes than of family structure [29]. Regardless of family type, lower supportive parenting and sensitivity foretold better father-reported child internalizing problems, while lower sensitivity and rough-and-tumble play quality, more negative parenting and parenting stress, and the male gender of the child predicted greater father-reported child externalizing problems [23]. Moreover, single fathers who felt more coparenting positive experiences within their families of origin exhibited lower levels of conflictual coparenting, which were, in turn, associated with better child attachment security [21].

Another area of concern would be the eventual exposure to stigma and discrimination of children and how this would affect their psychological well-being. Although the occurrence of behavioral problems is usually within the normal range, stigmatization (especially among single gay father-families) was related with children’s externalizing difficulties, even though the overall findings demonstrated that the use of surrogacy by single men had no unfavorable effects on the child overall health results [29,30]. In fact, children of single gay fathers through ARTs seemed to experience more intense microaggressions associated with their father’s sexual minority status which, in turn, predicted lower social skills and lower child–teacher relationship quality among school-aged children [31].

Irrespective of sexual orientation, single fathers via surrogacy could experience microaggressions since they encompass controversial features of family creation, namely, surrogacy conception, single parenthood, and, in the case of gay single fathers, same-sex orientation. Following this idea, a study examined the indirect effect of family-related microaggressions on observed sensitivity and rough-and-tumble play via rumination in single-father families with children (three to ten years) born through surrogacy [23]. Results showed that irrespective of their sexual orientation, single fathers who experienced more recurrent microaggressions also informed a higher propensity to ruminate in response to stress, and this was associated with lower sensitivity with their child.

When asking children about their views on their surrogacy origins, while most seem to show a clear understanding of their conception (“one woman donated an egg and an-other woman carried them in her tummy”), others presented some form of explanation that their fathers could not have had them on their own, generating some level of indifference (they did not think very often about it or did not show much interest about the subject), or feeling positive about it, expressing gratitude [32]. This reinforces the idea that, contrary to the belief that the children of single fathers via surrogacy find it difficult to deal with their origins, most are at peace with their origin trajectory.

Therefore, considering the results gathered in this mini review, it is safe to say that children born from surrogacy and raised by single fathers grow up and are as well psychosocially adjusted as their two-parent counterparts, when raised in emotionally secure and nurturing environments. Using surrogacy, single fathers are normalizing both surrogacy and single parenting by associating their families to previously existing family formation practices, managing their identity politics in an innovative manner, but, at the same time, distancing themselves from unconventional family models [33]. This, in turn, seems to be beneficial to their children by bringing their families more into alignment with dominant social expectations.

### 4.2. Implications for Research and Practice

Much of the investigation studying single-father families’ interactions and the psychosocial adjustment of children born via surrogacy, have demonstrated that these families are well adjusted and function well [7,8,9,10,11,12,13,14]. Far fewer studies have been implemented with children born via surrogacy than on families with children formed through other more recurrent procedures of reproduction [16,17,18,19,20,21,22,23]. Additionally, the current understanding of this these families is largely based on the experiences of white Italian, Israeli or American men who have the emotional and financial availability to pursue surrogacy. Even though this is largely influenced by the fact that there is a very limited number of countries where single men can access surrogacy, more research is needed to assess psychosocial adjustment and family relationships in other different sociocultural contexts.

Forthcoming studies should examine the dynamics of families formed by single fathers via surrogacy, specifically regarding the conceptualization of their sense of diversity, adversity, and family constellation, correctly informing all social players (policy makers, families, childcare providers), and therefore continue to challenge stigmatization and discrimination, and promoting positive coping mechanisms, resilience, and quality interaction to promote long-term positive adaptation.

Single-father families by surrogacy diverge from the traditional family in various ways; therefore, future studies should aim to understand how children and adolescents (since this age group has not been included in the studies reviewed) perceive their own family, examining adaptive and non-adaptive behavior triggered by eventual stigmatization. Additionally, variables that may protect children against those negative consequences should be studied, such as the contact with other similar families, supportive schools and communities, and legislations that should be favorable to the optimal functioning of single-father families created by surrogacy. The results of these studies have the potential to inform health, educational, social, and political stakeholders, and policies, and contribute to the expansion of empirical and theoretical knowledge in this field. This will be an opportunity to update professionals in various areas of intervention on the understanding of single fathers’ families created by surrogacy’s dynamics, focusing on the promotion of their children’s adjustment. In doing so, prejudice and stigma around the perception of surrogacy and single fatherhood as harmful for children’s developmental outcomes will not be supported by research, and this will allow practices to be based on the conclusion that positive psychosocial indicators are basically a function of relational processes rather than family types.

It is likely that children born to single-father families by surrogacy will be in contact with several contexts (extended family, school environments) where traditional views about family formation will be more prevalent. This, in turn, will eventually increase contact with stigmatization and disapproval, leading to lower self-esteem and negative developmental outcomes [22]. Hence, it is vital that these contexts can accommodate and provide positive and informed views and explanations regarding family diversity, i.e., that surrogacy and single fatherhood is not detrimental to children’s psychosocial adjustment. As children born to this type of families age, especially during adolescence, it will be likely that identity formation challenges, associated with their normal developmental tasks will arise; having the knowledge that their family type is well adjusted, this will increase the chances of resilience and positive coping when navigating adverse environments.

The results examined in this mini review will allow health professionals to acquire and develop informed intervention programs when working with this type of families, as well as actively participate in the information of legislators aimed at regulating access to ARTs by single men. This regulation should take into consideration that banning access single men to fertility treatments because it would be harmful to children and their psychosocial adjustment is completely unfounded.

## 5. Conclusions

Research with children of single-father families formed by surrogacy is still very insufficient, not only because of the lack of visibility and accessibility to ARTs by these families, but also because research with young children poses several challenges associated with their limited vocabulary, level of understanding and attention span. Still, the voices of children born to single-father families via surrogacy are beginning to be heard.

Overall, the studies here reviewed present evidence that these children show normal levels of psychosocial adjustment and can develop secure and positive relations with their fathers. Stigmatization associated with negative attitudes toward surrogacy (especially when intersections with sexual minority status occur) does appear to have an important role in the manifestation of behavioral problems, emphasizing the bidirectional nature of relations between parenting, the social environment, and child psychosocial adjustment.

Hence, single fathers can be just as capable, skilled, and resourceful as women at parenting, and the nonexistence of a mother does not partake any harmful consequences for the children’s psychosocial adjustment. It becomes clear that there is no empirical evidence to why single men, irrespective of their sexual orientation, should not be able to become fathers through surrogacy, with confirmed benefits for all those involved, especially their children.

It is of utmost importance that professional sectors of society and society at large stimulate and endorse access to ARTs, visibility, and respect of single-father families, as the mitigation of the exposure to adversity, stigma and discrimination would result on more positive results for single-father families, especially their children, created by surrogacy.

## Figures and Tables

**Table 1:** List of references from 2019 to 2022 investigating the psychosocial adjustment of children of single fathers created by surrogacy.

Authorship and Year	Country	Objective (s)	Number and Age of Participants	Short Description of Methods	Main Results
Shenkman; Carone; Mouton; d’Amore; Bos; 2022. [16]	Israel	To assess child externalizing problems among families formed by gestational surrogacy.	Gay fathers (*n* = 78) with children aged 3–10 years.	Multilevel modeling analyses to assess child externalizing problems, social support, negative and positive affect, self-efficacy, depression, and resilience.	Children of single fathers reported fewer externalizing problems and greater social support, when compared with straight couples.
Rubio; Rothblum; Bergman; Katuzny; 2019. [17]	United States of America	To assess the behavioral functioning of children conceived via gestational surrogacy.	Children (*n* = 68) of gay fathers aged 3–10.	The Achenbach Child Behavior Checklist was used.	Surrogacy children had significantly lower scores of anxiety and depression than the national database.
Carone; Baiocco; Lingiardi; Kerns; 2019. [18]	Italy	To assess child attachment security in gay surrogacy families.	Children (*n* = 33) of gay fathers aged between 8 and 11.8 years.	Data were collected from father-child observed interactions, interviews, and questionnaires.	Children perceived high levels of attachment security not differing from normative scores of children with straight parents.
Carone; Barone; Manzi; Baiocco; Lingiardi; Kerns; 2020. [19]	Italy	To examine influences of child attachment security and parental scaffolding in children’s exploration of their surrogacy origins.	Surrogacy children (*n* = 30) born to gay fathers aged between 6 and 12 at time 1, and 7.5 and 13.5 at time 2.	Longitudinal study of father-child dyads who participated in a videotaped conversation, and children were also administrated a security attachment questionnaire.	Children who presented higher levels of attachment security communicated greater exploration of their surrogacy origins.
Carone; Barone; Lingiardi; Baiocco; Brodzinsky; 2021. [20]	Italy	To assess factors associated with behavioral adjustment among school-aged children of single father through surrogacy.	Children of gay single fathers (*n* = 31) and children of heterosexual single fathers (*n* = 28). All children were aged 6–12 years.	Measures included children’s perceptions of self-worth and their fathers report on behavioral adjustment.	No differences were found across family groups in behavioral outcomes, with children demonstrating high levels of self-worth and low levels of behavioral problems.
Carone; 2022. [21]	Italy	To assess family alliance perceptions and intergenerational transmission of coparenting in single-father families through surrogacy.	Children of gay single fathers (*n* = 31) and children of heterosexual single fathers (*n* = 28). All children were aged 6–12 years.	Measures of coparenting in their family of origin, observed family alliance and child attachment security were used. Additionally, father-child interactions were videotaped.	Families did not differ in family alliance based on father’s sexual orientation, and single fathers who experienced greater coparenting quality in their families of origin experienced less conflict and greater attachment security.
Carone; Baiocco; Lingiardi; Barone; 2020. [22]	Italy	To examine father-child relationship and child psychological adjustment in single-father families.	Gay single-father families (*n* = 35); heterosexual single-father families (*n* = 35), gay two-father surrogacy families (*n* = 45); and heterosexual two-parent IVF families (*n* = 45). All children were aged 3–10 years.	Standardized questionnaires and interviews were used, as well as video recorded observational tasks with children.	The psychological adjustment of children born to gay and heterosexual fathers via surrogacy is more a function of family processes than family structure.
Carone; Lingiardi; Baiocco; Barone; 2021. [23]	Italy	To assess sensitivity and rough-and-tumble play in gay and heterosexual single-father families.	Gay (*n* = 35) and heterosexual (*n* = 30) single-father families (*n* = 65) with children aged 3–10 years.	Measures included experiences of microaggression and observed sensitivity and rough-and-tumble play through rumination.	The experience of microaggressions was linked to lower sensitivity with their child, but not rough-and-tumble play quality.

## Data Availability

Not applicable.

## References

[1] Settles B.H., Steinmetz S. (2009). Concepts and Definitions of Family for the 21st Century.

[2] Pereira H., Beatriz C. (2022). Promoting Social Visibility for Single-Father Families Created by Surrogacy. Fam. Soc..

[3] Krajewska A., Cahill-O’Callaghan R. (2020). When a Single Man Wants to Be a Father: Revealing the Invisible Subjects in the Law Regulating Fertility Treatment. Soc. Leg. Stud..

[4] Liu J., Bice S. (2021). Surrogacy, social policy, and economic development. Asian J. Adv. Med. Sci..

[5] U.S. Census Bureau (2017). Facts for Features: Father’s Day. https://www.census.gov/newsroom/facts-for-features/2017/cb17-ff12-fathers-day.html?#.

[6] Eurostat (2019). People in the EU—Statistics on Household and Family Structures. https://ec.europa.eu/eurostat/statistics-explained/index.php?title=People_in_the_EU_-_statistics_on_household_and_family_structures#Single-person_households.

[7] Fantus S. (2021). Experiences of gestational surrogacy for gay men in Canada. Cult. Health Sex..

[8] Carone N., Baiocco R., Lingiardi V. (2017). Single fathers by choice using surrogacy: Why men decide to have a child as a single parent. Hum. Reprod..

[9] Maya T., Adital B.-A. (2021). Experiences and Meanings of Surrogate Pregnancy among Gay Israeli Men Who Become Parents through Overseas Surrogacy. J. Homosex..

[10] Bergman K., Rubio R.J., Green R.J., Padrón E. (2010). Gay Men Who Become Fathers via Surrogacy: The Transition to Parenthood. J. GLBT Fam. Stud..

[11] Maya T., Adital B.-A. (2021). Single gay fathers via surrogacy: The dialectics between vulnerability and resilience. J. Fam. Stud..

[12] Igreja A.R., Ricou M. (2019). Surrogacy: Challenges and ambiguities. New Bioeth..

[13] Jones C., Zadeh S., Jadva V., Golombok S. (2022). Solo Fathers and Mothers: An Exploration of Well-Being, Social Support and Social Approval. Int. J. Environ. Res. Public Health.

[14] Maya T., Segal-Engelchin D. (2022). The Social Experiences of Single Gay Fathers in Israel: An Intersectional Perspective. Int. J. Environ. Res. Public Health.

[15] Page M.J., McKenzie J.E., Bossuyt P.M., Boutron I., Hoffmann T.C., Mulrow C.D., Shamseer L., Tetzlaff J.M., Akl E.A., Brennan S.E. (2021). The PRISMA 2020 statement: An updated guideline for reporting systematic reviews. Syst. Rev..

[16] Shenkman G., Carone N., Mouton B., Shenkman G., Carone N., Mouton B., d’Amore S., Bos H.M.W. (2022). Assisted Conception Socialization Self-Efficacy Among Israeli Lesbian, Gay, and Heterosexual Parent Families and its Association with Child Externalizing Problems. J. Child Fam. Stud..

[17] Rubio R., Rothblum E.D., Bergman K., Katuzny K. (2019). Gay Fathers by Surrogacy: Prejudice, Parenting, and Well-being of Female and Mala Children. Psychol. Sex. Orientat. Gend. Divers..

[18] Carone N., Baiocco R., Lingiardi V., Kerns K. (2020). Child attachment security in gay father surrogacy families: Parents as safe havens and secure bases during middle childhood. Attach. Hum. Dev..

[19] Carone N., Barone L., Manzi D., Baiocco R., Lingiardi V., Kerns K. (2020). Children’s Exploration of Their Surrogacy Origins in Gay Two-Father Families: Longitudinal Associations with Child Attachment Security and Parental Scaffolding During Discussions About Conception. Front. Psychol..

[20] Carone N., Barone L., Lingiardi V., Baiocco R., Brodzinsky D. (2021). Factors associated with behavioral adjustment among school-age children of gay and heterosexual single fathers through surrogacy. Dev. Psychol..

[21] Carone N. (2022). Family Alliance and Intergenerational Transmission of Coparenting in Gay and Heterosexual Single-Father Families through Surrogacy: Associations with Child Attachment Security. Int. J. Environ. Res. Public Health.

[22] Carone N., Baiocco R., Lingiardi V., Barone L. (2020). Gay and heterosexual single father families created by surrogacy: Father–child relationships, parenting quality, and children’s psychological adjustment. Sex. Res. Soc. Policy A J. NSRC.

[23] Carone N., Lingiardi V., Baiocco R., Barone L. (2021). Sensitivity and rough-and-tumble play in gay and heterosexual single-father families through surrogacy: The role of microaggressions and fathers’ rumination. Psychol. Men Masc..

[24] Rodríguez-Fernández A., Ramos-Díaz E., Madariaga J.M., Arrivillaga A., Galende N. (2016). Steps in the construction and verification of an explanatory model of psychosocial adjustment. Eur. J. Educ. Psychol..

[25] Bowlby J. (1969/1982). Attachment, and Loss: Attachment.

[26] Golombok S., Murray C., Jadva V., MacCallum F., Lycett E. (2004). Families created through surrogacy arrangements: Parent-child relationships in the 1st year of life. Dev. Psychol..

[27] Bos H., van Balen F. (2010). Children of the new reproductive technologies: Social and genetic parenthood. Patient Educ. Couns.

[28] Golombok S., Readings J., Blake L., Casey P., Marks A., Jadva V. (2011). Families created through surrogacy: Mother-child relationships and children’s psychological adjustment at age 7. Dev. Psychol..

[29] Golombok S., Blake L., Slutsky J., Raffanello E., Roman G.D., Ehrhardt A. (2018). Parenting and the Adjustment of Children Born to Gay Fathers Through Surrogacy. Child. Dev..

[30] Carone N., Lingiardi V., Chirumbolo A., Baiocco R. (2018). Italian gay father families formed by surrogacy: Parenting, stigmatization, and children’s psychological adjustment. Dev. Psychol..

[31] Carone N., Innocenzi E., Lingiardi V. (2022). Peer Microaggressions and Social Skills among School-Age Children of Sexual Minority Parents through Assisted Reproduction: Moderation via the Child–Teacher Relationship. J. Youth Adolesc..

[32] Carone N., Baiocco R., Manzi D., Antoniucci C., Caricato V., Pagliarulo E., Lingiardi V. (2018). Surrogacy families headed by gay men: Relationships with surrogates and egg donors, fathers’ decisions over disclosure and children’s views on their surrogacy origins. Hum. Reprod..

[33] Smietana M. (2016). Families Like We’d Always Known? Spanish Gay Fathers’ Normalization Narratives in Transnational Surrogacy. Assisted Reproduction Across Borders.

